# Knowledge, attitudes and practices (KAP) towards COVID-19 among Palestinians during the COVID-19 outbreak: A cross-sectional survey

**DOI:** 10.1371/journal.pone.0244925

**Published:** 2021-01-05

**Authors:** Nouar Qutob, Faisal Awartani

**Affiliations:** Department of Health Sciences, Faculty of Graduate Studies, Arab American University, Ramallah, Palestine; National Institute for Infectious Diseases Lazzaro Spallanzani-IRCCS, ITALY

## Abstract

Coronavirus disease 2019 (COVID-19) is a highly contagious illness that spreads rapidly through human-to-human transmission. On March 5, the government of Palestine declared a state of emergency in order to curb the spread of the virus, a declaration that it extended for a fifth time on July 5^th^. The degree to which a population complies with corresponding safety measures is surely affected by the people’s knowledge, attitudes and practices (KAP) towards the disease. To explore this hypothesis, we gathered data from 1,731 Palestinians between April 19^th^and May 1^st^, 2020 through a KAP questionnaire. The participant pool represented a stratified sample of Palestinians living across a number of governorates in the Gaza Strip and the West Bank, with 36.5% from Gaza and (63.5%) from the West Bank. Gender was almost equally distributed within the sample with (51%) men respondents and (49%) women respondent. The questionnaire included 17 questions about participants’ knowledge and awareness of COVID–19, 17 questions regarding the safety measures they had taken in the wake of the outbreak and 3 questions asking them to assess the efficacy of the government’s response to the pandemic. Our data shows that 79% of the respondents have good awareness about transmission of the virus, 55.6% were knowledgeable of the symptoms exhibited by an infected individual, 81% were aware of the preventative measures and 82% demonstrated awareness of the risk groups. Most participants complied with preventative measures (77%) and 62% the study participants agreed that stricter measures have to be enforced by the government to limit the spread of the virus. Our study revealed that younger participants and people with higher educational level demonstrated more awareness of the virus. Also, Women were reported to be more aware of preventative measures and to have complied more with good practices. We report that residents of the West Bank have complied more with the right practices when compared to residents of Gaza. Based on the results of this study, we conclude that health education programs aimed at improving the public’s understanding of COVID-19 are important in helping the population maintain appropriate practices and should be target people with lower educational level, and that findings such as those discussed in this report may provide valuable feedback to lawmakers working to stop the spread of the virus.

## Introduction

Coronavirus disease 2019, known as COVID-19, is an infectious respiratory disease caused by novel coronavirus SARS-CoV-2 [1]. Since its emergence in Wuhan, China in December 2019 [2], SARS-CoV-2 has spread rapidly around the globe, ultimately being declared by the World Health Organization (WHO) as a global pandemic [3, 4]. Daily, new cases and deaths are being reported worldwide [5].

On March 5^th^, the government of Palestine declared an emergency period after seven Palestinians tested positive for (SARS-CoV-2) in Bethlehem. In an effort to fight the coronavirus, educational institutions, tourist attractions, mosques, churches and parks were closed for a duration of one month. On March 18^th^, a curfew was declared obliging the public to abide by social distancing guidelines and to stay in quarantine except in cases of emergency. On May 4^th^, this state of emergency was extended by one month for a third time.

In the Palestinian Territories, 848 cases and 4 deaths have been reported by the Palestinian Ministry of Health (MOH) as of May 15^th^, 2020. On May 25^th^, the restrictions were eased following a decline in cases and a reduction of the rate of positive tests in Palestinian workers returning from Israeli areas. Seroprevalence, as measured up to July 2020 remains low [6]. However, cases surged again during July. On the 3^rd^ of July, a ten-day complete lockdown was declared across the entire West Bank. On the 12^th^ of July, a complete lockdown for five days was declared in Hebron, Bethlehem, Ramallah and Nablus governorates. A state of emergency was extended for a tenth time on October 1^st^. Cases continued to rise till it reached 112,035 confirmed cases and 922 deaths in December.

Adherence to mandated protective measures is essential in stopping the spread of the virus but is also dependent on the population’s overall knowledge, attitudes and awareness, according to KAP theories [7–9].

Studies conducted during the SARS outbreak in 2003 suggest that knowledge, attitudes and practices towards viruses are associated with emotions among populations and can indeed complicate attempts to prevent the spread of the virus [10, 11]. In order to craft effective policy regarding the management of COVID-19, it is important that health officials have the tools they need to gauge the public’s awareness of, and attitude towards, the disease. To this end, we conducted a cross-sectional survey designed to assess KAP towards COVID-19 among Palestinians. Our findings emphasize the need to investigate the KAP towards COVID-19 among Palestinians residing in the West Bank and Gaza.

## Materials and methods

### Participants

Ethics approval for the research was obtained from the Helsinki committee (PHRC/HC/718/20) Participants recruited in the study gave verbal consent forms prior to participation in the study voluntary agreeing to participate in research.

This cross-sectional study was conducted between April 19th and May 1^st^, 2020. Data was collected through Computer Assisted Phone Interviewing (CATI) using resident phone numbers available through the Reach Calling Center. A data entry program developed by Alpha International Research technical staff on the online platform KoBo was provided to Reach Center callers for data collection. The resulting data was monitored on a daily basis.

The target population for this study was Palestinians living in the West Bank and Gaza Strip with ages more than 15 years old. In total, we received completed questionnaires from 1,731 participants, of whom 49.3% were women and 50.7% were men. 36.5% of respondents were from Gaza and 63.5% were from the West Bank, including participants from different areas of residency; 74.5% of respondents were living in cities, 19.1% in villages and 6.8% in camps. 53.8% held a Bachelor’s degrees or higher.

### Measures

The questionnaire consisted of two parts: demographics and KAP. Demographic variables included gender, age, locality, academic achievement, employment, and employment sector. The KAP section included 34 questions about knowledge of COVID-19 (Table 1; Q1-18), including 10 questions regarding virus transmission (Q1:1–10), 9 regarding symptoms (Q1:11:1–9), 9 regarding who is included in high and low risk groups (Q1:12:1–9) and 6 regarding disease prevention and control (Q1:13–18). Attitudes towards COVID-19 were measured by 3 questions (Table 1; Q2:1–3) that asked respondents about their confidence that COVID-19 would successfully be controlled, their confidence that Palestine would win the battle against COVID-19 and if they believe that stricter measurements should be applied. The assessment of practices was composed of seven questions about the practices and behaviors of participants (Table 1, Q3:1–7).

**Table 1:** Questions used to test the participants knowledge, attitudes and practices.

	Questions
Q1:1	The virus that causes COVID-19 spreads via respiratory droplets
Q1:2	The virus that causes COVID-19 is thought to spread mainly from person to person
Q1:3	An individual who had the disease can spread the illness to others?
Q1:4	An individual in quarantine can spread the illness to others?
Q1:6	The virus that causes corona (Covid-19) can spread through surfaces
Q1:8	Infected people cannot transmit the virus if they do not exhibit fever
Q1:9	Warm weather would stop the outbreak
Q1:10	There is no effective cure for COVID-19
Q1:11:1	Is fever a symptom of Covid-19
Q1:11:2	Is sneezing a symptom of Covid-19
Q1:11:3	Is dry cough a symptom of Covid-19
Q1:11:4	Is vomiting a symptom of Covid-19
Q1:11:5	Is runny nose a symptom of Covid-19
Q1:11:6	Do you consider blurred vision as a symptom of Covid-19
Q1:11:7	Is Myalgia symptom of Covid-19
Q1:11:8	Is stuffy nose a symptom of Covid-19
Q1:11:9	Is frequent urination a of Covid-19
Q1:12:1	Diabetics are in high risk categories
Q1:12:2	People with respiratory illness are in high risk categories
Q1:12:3	People with heart condition are in high risk categories
Q1:12:4	Elderly (70 years or more) are in high risk categories
Q1:12:5	People at age category (50 to 69 years old) are in high risk categories
Q1:12:6	People at age category (30 to 49 years old) are in high risk categories
Q1:12:7	Young Adults (20–29 years old) are in high risk categories
Q1:12:8	Teenagers (10–20 years old) are in high risk categories
Q1:12:9	Children under 10 years old are in high risk categories
Q1_13	Individuals can wear general medical masks to prevent the infection by the COVID-19 virus
Q1_14	It is not necessary for children and young adults to take preventative measures against the virus
Q1_15	individuals should avoid going to crowded places as a preventative measure against the virus
Q1_16	Isolation is an effective way to reduce the spread of the virus.
Q1_17	An individual who has been in close contact with an infected person should be in quarantine for 14 days
Q1_18	COVID-19 is caused by a coronavirus called SARS-CoV-2
Q2:1	Do you agree that the infection rate can be controlled in Palestine?
Q2:2	Do you have confidence the Palestinian government will be able to stop the spread of the disease in Palestine?
Q2:3	Do you believe that should impose a) stricter measurements b) less strict measures c) keep the situation as it is
Q3:1	During the last week, have you been in a crowded place?
Q3:2	During the last week, have you been to your work place?
Q3:3	During the last week, have you visited your neighbors?
Q3:4	During the last week, have you visited any of your relatives and/or friends?
Q3:5	During the last week, have you worn gloves? masks when leaving home?
Q3:6	During the last week, have you kept a distance of at least two between you and others?
Q3:7	During the last week, have you cleaned your hands regularly by rubbing an alcohol-based hand sanitizer or washing them with soap and water?

### Data analysis

Data analysis was conducted using the statistical software (IBM SPSS 23.0) to produce a preliminary cross-tabulation of the study variables with the background indicators. This statistical report is comprised of cross-tabulations of all the study variables representing knowledge, attitudes and practice as well as the demographic characteristics of respondents. We used ANOVA and Chi-Square to test bivariate relationships of study variables across the different background variables. ANOVA tests the relationship between a quantitative dependent variable and a qualitative independent variable, while Chi-Square test is used to test the relationship between a qualitative dependent variable and a qualitative independent variable.

The questions were answered on a true/false/I don’t know basis. A correct answer was assigned 1 point and an incorrect answer or an ‘I don’t know’ answer was assigned 0 points. Scores were calculated indicated in Table 2.

**Table 2:** Definition of the five scores used in the analysis.

Score	Definition
General knowledge score about virus transmission	The percentage of correct answers of questions Q1_1 through Q1_10
General knowledge score about COVID-19 symptoms	The percentage of correct answers of questions Q1_11_1 through Q1_11_9
General knowledge score about high and low risk groups	The percentage of correct answers for questions Q1_12_1 through Q1_12_9
General knowledge score about proper health measures	The percentage of correct answers for questions Q1_13 through Q1_18.
General practice of good hygiene and social distancing.	The percentage of correct answers of Q3_8 through Q3_12.

## Results

**Fig 1:**
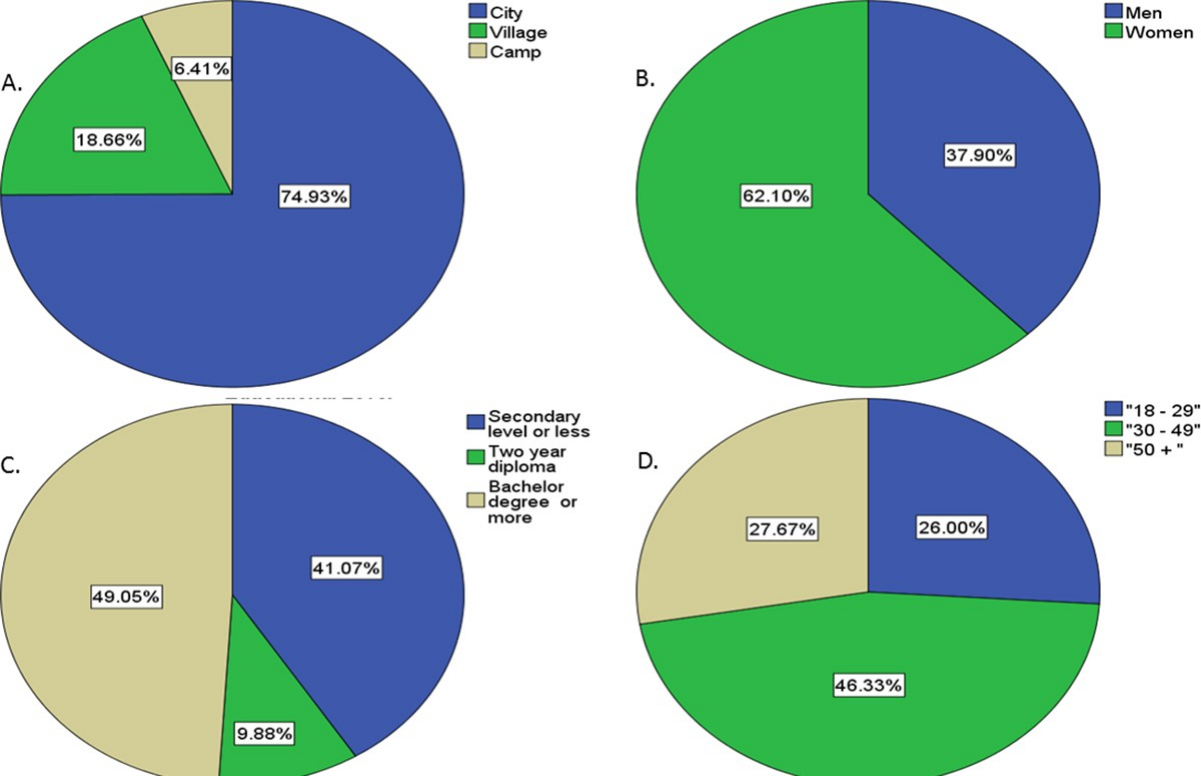
(a) Geographical region, (b) gender, (c) educational level and (d) age group distribution of the 1,731 participants between April 19^th^and May 1^st^, 2020 in the West Bank and Gaza.

A total of 1,731 Palestinians living across a number of governorates in the Gaza Strip and the West Bank, with 36.5% from Gaza and (63.5%) from the West Bank participated in the study. Gender was almost equally distributed within the sample with (51%) men respondents and (49%) women respondents (Fig 1).

### Knowledge of participants towards Covid-19

Our data shows good awareness of respondents about transmission of the virus (79%), symptoms exhibited by an infected individual (55.6%), preventative measures (81%) and risk groups (82%). We noted that the symptoms knowledge score was the lowest compared to other knowledge scores across the different categories (Table 3 and S1–S4 Tables).

**Table 3:** Knowledge scores across geographical regions, gender, educational levels and age groups. The study included 1,731 participants between April 19^th^and May 1^st^, 2020 in the West Bank and Gaza.

			N	Mean	Std. Deviation	95% Confidence Interval for Mean		P-value ANOVA Test
						Lower Bound	Upper Bound	
Transmission of virus knowledge score	Geographical Location	West Bank	1120.0	78.8	14.0	78.0	79.7	0.079
		Gaza Strip	611.0	80.0	12.6	79.0	81.0	
	Gender	Men	656.0	79.5	13.5	78.5	80.6	0.539
		Women	1075.0	79.1	13.5	78.3	79.9	
	Educational Level	Secondary Education or less	711.0	77.1	13.3	76.1	78.1	0.000
		Two-year diploma	171.0	78.8	13.4	76.8	80.8	
		Bachelor degree or more	849.0	81.2	13.5	80.3	82.1	
	Age group	18–29	450.0	80.6	13.9	79.4	81.9	0.008
		30–49	802.0	79.3	13.3	78.4	80.2	
		50 +	479.0	77.9	13.5	76.7	79.1	
		Total	1731.0	79.3	13.5	78.6	79.9	
Symptoms knowledge score	Geographical Location	West Bank	1120.0	55.6	18.2	54.5	56.7	0.915
		Gaza Strip	611.0	55.5	17.2	54.2	56.9	
	Gender	Men	656.0	54.8	18.5	53.4	56.3	0.179
		Women	1075.0	56.0	17.4	55.0	57.1	
	Educational Level	Secondary Education or less	711.0	52.3	18.0	51.0	53.7	0.000
		Two-year diploma	171.0	56.1	18.0	53.4	58.9	
		Bachelor degree or more	849.0	58.2	17.2	57.0	59.3	
	Age group	18–29	450.0	57.4	17.9	55.7	59.0	0.000
		30–49	802.0	56.2	17.3	55.0	57.4	
		50 +	479.0	52.9	18.3	51.3	54.6	
		Total	1731.0	55.6	17.8	54.7	56.4	
High risk group knowledge score	Geographical Location	West Bank	1120.0	81.3	16.6	80.4	82.3	0.001
		Gaza Strip	611.0	84.0	14.7	82.8	85.1	
	Gender	Men	656.0	82.3	15.7	81.1	83.5	0.888
		Women	1075.0	82.2	16.1	81.3	83.2	
	Educational Level	Secondary Education or less	711.0	81.0	16.4	79.8	82.2	0.012
		Two-year diploma	171.0	81.9	15.8	79.6	84.3	
		Bachelor degree or more	849.0	83.4	15.6	82.3	84.4	
	Age group	18–29	450.0	81.9	16.2	80.4	83.4	0.399
		30–49	802.0	82.8	15.9	81.7	83.9	
		50 +	479.0	81.7	15.9	80.3	83.1	
		Total	1731.0	82.3	16.0	81.5	83.0	
Health measures knowledge score	Geographical Location	West Bank	1120.0	82.1	14.8	81.2	83.0	0.139
		Gaza Strip	611.0	81.0	14.5	79.8	82.1	
	Gender	Men	656.0	80.8	15.1	79.6	81.9	0.046
		Women	1075.0	82.2	14.5	81.4	83.1	
	Educational Level	Secondary Education or less	711.0	81.3	15.3	80.2	82.4	0.171
		Two-year diploma	171.0	80.3	14.8	78.1	82.5	
		Bachelor degree or more	849.0	82.3	14.2	81.4	83.3	
	Age group	18–29	450.0	81.7	13.5	80.4	82.9	0.762
		30–49	802.0	81.9	15.0	80.9	83.0	
		50 +	479.0	81.3	15.2	79.9	82.7	
		Total	1731.0	81.7	14.7	81.0	82.4	

N: number of respondents; Std Deviation: standard deviation.

Residents of the West Bank demonstrated to be as knowledgeable about the transmission of the virus, the symptoms exhibited by an infected individual as well as preventative measures as residents of Gaza (p-value = 0.079, 0.915, 0.14 respectively). In contrast, individuals residing in the West Bank demonstrated to be more knowledgeable about risk groups when compared to those residing in Gaza (p-value = 0.001) (Table 3).

Younger participants demonstrated more knowledge about transmission as well as symptoms exhibited by infected individuals than older groups (p-value = 0.0080, <0.001, respectively). No difference was reported in the awareness about risk groups and the preventative measures across the different age groups (p- value = 0.399, 0.762, respectively) (Table 3).

Respondents with higher educational level demonstrated more awareness of transmission, symptoms and risk group when compared to groups with lower educational levels (p-value = 0.000, 0.000, 0.012 respectively). Knowledge about preventive measures did not show significant difference across the different educational levels (p-value = 0.171) (Table 3).

Women were reported to be more aware of preventative measures (p-value = 0.046). No significant difference was noted between women and men in terms of their awareness to the transmission, symptoms and risk groups (p-value = 0.56, 0.179, 0.888) (Table 3).

### Attitudes of participants

In general, the majority of the study participants agreed that stricter measures have to be enforced by the government to limit the spread of the virus (62.4%). Perceptions of participants towards the preventative were significantly different across geographical regions, gender and age group (p-value <0.001, 0.009, <0.001 respectively). However, no difference in attitudes between respondents of different educational levels was noted (Table 4).

**Table 4:** Attitudes scores across geographical regions, gender, educational levels and age groups. The study included 1,731 participants between April 19^th^and May 1^st^, 2020 in the West Bank and Gaza.

			
		stricter measures	less strict measures	The situation remains the same	Total	Chi-Square P-value
Geographical Location	West Bank	57.9%	7.2%	34.9%	100.0%	0.0000
	Gaza Strip	70.7%	4.1%	25.2%	100.0%	
Gender	Men	59.5%	8.2%	32.3%	100.0%	0.0090
	Women	64.2%	4.8%	31.0%	100.0%	
Educational Level	Secondary level or less	60.1%	5.8%	34.2%	100.0%	0.1700
	Two-year diploma	59.1%	7.6%	33.3%	100.0%	
	Bachelor degree or more	65.0%	6.1%	28.9%	100.0%	
	Secondary level or less	60.1%	5.8%	34.2%	100.0%	
Age group	18–29	70.7%	2.9%	26.4%	100.0%	0.0000
	30–49	61.0%	6.9%	32.2%	100.0%	
	50 +	57.0%	7.9%	35.1%	100.0%	
	18–29	70.7%	2.9%	26.4%	100.0%	
	Total	62.4%	6.1%	31.5%	100.0%	

The majority of Palestinians reported to be optimistic that COVID-19 infection rate can be controlled. More women showed optimism than men (p-value = 0.01). Respondents with higher level education were less optimistic than those with lower educational level (p-value <0.001). The majority of Palestinians reported confidence in the Palestinian government’s capability to stop the spread of the virus (94%). The data shows statistically significant differences in perception across geographical regions, gender, educational level and age groups (p-value =, 0.003, <0.001, 0.003, 0.01, respectively) (Tables 5 and 6).

**Table 5:** Attitude of the study participants towards the control of infection rate in Palestine scores across geographical regions, gender, educational levels and age groups. The study included 1,731 participants between April 19^th^and May 1^st^, 2020 in the West Bank and Gaza.

Do you agree that the infection rate can be controlled in Palestine?						
		Agree	Disagree	Don't know	Total	Chi-Square P-value
Geographical Location	West Bank	89.30%	7.80%	2.90%	100.00%	0.28
	Gaza Strip	91.70%	5.90%	2.50%	100.00%	
Gender	Men	86.60%	9.60%	3.80%	100.00%	0.001
	Women	92.30%	5.60%	2.10%	100.00%	
Educational Level	Secondary level or less	93.80%	4.10%	2.10%	100.00%	0.000
	Two-year diploma	89.50%	8.20%	2.30%	100.00%	
	Bachelor degree or more	87.20%	9.40%	3.40%	100.00%	
Age Group	18–29	89.80%	8.20%	2.00%	100.00%	0.42
	30–49	89.80%	7.40%	2.90%	100.00%	
	50 +	91.00%	5.60%	3.30%	100.00%	

**Table 6:** Attitudes of the study participants towards the ability of the Palestinian government to stop the spread of the disease in Palestine scores across geographical regions, gender, educational levels and age groups. The study included 1,731 participants between April 19^th^and May 1^st^, 2020 in the West Bank and Gaza.

Do you have confidence the Palestinian government will be able to stop the spread of the disease in Palestine?						P-value Chi-Square test
			Yes	No	Total	
Geographical Location	West Bank	Row N %	93.0%	7.0%	100.0%	0.003
	Gaza Strip	Row N %	96.6%	3.4%	100.0%	
Gender	Men	Row N %	91.8%	8.2%	100.0%	0.000
	Women	Row N %	95.8%	4.2%	100.0%	
	Total	Row N %	94.3%	5.7%	100.0%	
Educational Level	Secondary level or less	Row N %	96.2%	3.8%	100.0%	0.003
	Two-year diploma	Row N %	95.9%	4.1%	100.0%	
	Bachelor degree or more	Row N %	92.3%	7.7%	100.0%	
Age Group	18–29	Row N %	91.6%	8.4%	100.0%	0.01
	30–49	Row N %	94.8%	5.2%	100.0%	
	50 +	Row N %	96.0%	4.0%	100.0%	

N: number of respondents.

### Practices of participants

Our data shows that respondents in general have complied with preventative measures (77%).

Residents of the West Bank were seen to have complied more with the right practices when compared to residents of Gaza (p-value < 0.001). Also, women were shown to be complying more with the preventative practices then men (p-value <0.001). In contrast, our data shows no significant differences in behaviors across the different age groups and educational levels (P-Value = 0.192,0.901, respectively) (Table 7 and S5 Table).

**Table 7:** Practice scores across scores across geographical regions, gender, educational levels and age groups. The study included 1,731 participants between April 19^th^and May 1^st^, 2020 in the west bank and Gaza.

			N	Mean	Std. Deviation	95% Confidence Interval for Mean		P-value ANOVA Test
						Lower Bound	Upper Bound	
Practice Score	Geographical Location	West Bank	1120.0	79.7	15.5	78.8	80.6	0.000
		Gaza Strip	611.0	70.8	19.2	69.2	72.3	
	Gender	Men	656.0	74.2	19.4	72.7	75.7	0.000
		Women	1075.0	78.0	16.0	77.0	78.9	
	Educational Level	Secondary Education or less	711.0	76.5	17.5	75.2	77.8	0.901
		Two-year diploma	171.0	76.0	17.2	73.4	78.6	
		Bachelor degree or more	849.0	76.7	17.5	75.5	77.9	
	Age group	18–29	450.0	76.7	17.7	75.1	78.4	0.192
		30–49	802.0	75.8	17.5	74.6	77.0	
		50 +	479.0	77.6	17.1	76.1	79.1	
		Total	1731.0	76.5	17.5	75.7	77.4	

N: number of respondents; Std Deviation: standard deviation.

## Discussion

The purpose of this study was to estimate the general level of awareness, practice and attitude towards COVID-19 among Palestinians residing the West Bank and Gaza Strip. In general, we found that the majority of participants had a good base of awareness about the virus which may have led to the respondents’ compliance with preventative measures and the positive attitude towards the COVID-19 pandemic.

The respondents demonstrated good awareness of transmission of the virus, the preventative measures as well as the risk groups, which is consistent with other studies conducted worldwide [11, 12]. Only 55.6% of the respondents demonstrated good awareness of the symptoms exhibited by an infected individual. This may be because at the time of data collection, the virus was new and the residents were learning more about the symptoms every day.

In general residents of the West Bank demonstrated similar COVID-19 awareness as residents of Gaza Strip; however, individuals residing in the West Bank demonstrated to be more aware about risk groups when compared to those residing in Gaza Strip, which may be attributed to restricted movement and low income. Studies conducted in Saudi Arabia [13], Egypt [14], China [15], USA [7] showed that respondents with higher income demonstrated more awareness of the virus. Even though residents of Gaza demonstrated similar awareness of the preventative measures as residents of the West Bank, they have complied less with the right practices. Therefore, government in Gaza should make more efforts to enforce the measures using different communication channels.

Our study shows that women demonstrated more COVID-19 awareness compared to men, unlike what was reported in a previous study in Saudi Arabia [16], but consisted with a study in the USA [17]. Also, women were shown to have complied more with the preventative practices then men. This can be explained by different gender-related activities and family roles or maybe a consequence of females experiencing higher levels of stress, anxiety, and depression [18]. A survey conducted by KFF found that women are more likely than men to worry about the negative consequences of the pandemic and to report mental health effects from worrying about coronavirus. The study reported that women stay at home instead of going to work, school, or other regular activities [19]. In this respect, women tend to maintain social distancing and play an essential role in public health management [20].

Moreover, respondents with higher educational level demonstrated more awareness of the virus. This is consistent with previous studies in the Arab World that reported that high education level is an important predictor of greater COVID-19 knowledge scores [7, 13–15]. Nevertheless, even though respondents with higher educational levels demonstrated more awareness of the virus, they had a more positive attitude against COVID-19 and they did not comply more with the good practices compared to respondents with lower educational levels, which is inconsistent with a study reporting the impact of awareness on good practice [21].

Our data shows that the younger participants demonstrated more knowledge about transmission as well as symptoms exhibited by infected individuals than the older generation, an observation which could be explained by the increased usage of different social media channels compared to the older group. A result consistent with a previous study [16].

Most participants had confidence that COVID-19 would be eliminated and expressed certainty that the Palestinian government is able to control the spread of the disease, This finding is consistent with recent studies conducted, where the majority of participants were convinced that the disease is curable and that their country will combat it [13, 22], but in contrast with other findings that suggest people tend to express negative emotions during a pandemic that could affect their attitude [23]. This optimism could be a result of the strictness of the disease control measures taken by Palestinian officials, which enhances people’s confidence in their approach. This attitude could also be attributed to positive practices reported by participants.

Overall, the score for the practice section was high (81%) for most participants. These practices could be attributed to the restriction of movement implemented by the government, a practice previously described to be an effective measure against the spread of Covid-19 [24] and the fact that participants demonstrated strong knowledge about how COVID-19 is transmitted. Our study shows that higher COVID-19 awareness was found to be significantly associated with a lower likelihood of having negative attitudes about the government’s handling of the pandemic and practicing potentially dangerous behaviors. This is consistent with a previous study reporting that lack of awareness contributes to undesirable attitudes [21].

These findings clearly indicate the importance of improving residents’ COVID-19 knowledge via health education, as this may both improve their outlook and result in safer personal practices. The strength of this study lies in its large sample size which was recruited during a peak of the COVID-19 outbreak. speculate that our findings may overestimate the extent of the population’s disease related knowledge. However, one limitation of the study is that 74.5% of the participants come from the cities and are therefore more likely to come from privileged groups that have more COVID-19 awareness. To control for this factor, future KAP studies should include a greater proportion of participants from villages and camps.

To the best of our knowledge, this is the first cross-sectional survey done in Palestine to examine the KAP towards COVID-19 among Palestinians. We hope that the study will facilitate the implementation of effective policy by enabling health officials to better understand the awareness, knowledge and attitudes held by the Palestinian population towards COVID-19.

## Supporting information

S1 Table Knowledge of respondents about transmission of the virus that causes COVID-19 (Q1_1 through Q1_10). (DOCX)

S2 Table Knowledge of respondents about Covid-19 symptoms (Q1_11_1 through Q1_11_9). (DOCX)

S3 Table Knowledge of respondents about high risk groups (Q1_12_1 through Q1_12_9). (DOCX)

S4 Table Knowledge of respondents about preventative measures (Q1_13 through Q1_18). (DOCX)

S5 Table Practices of respondents towards preventative measures (Q3_8 through Q3_12). (DOCX)
