# Supplementary material for: Knowledge, attitudes and practices (KAP) towards COVID-19 among Palestinians during the COVID-19 outbreak: A cross-sectional survey
Source: PLoS One. 2021 Jan 5;16(1):e0244925. doi: 10.1371/journal.pone.0244925 (PMC7785223; doi:10.1371/journal.pone.0244925)
Supplement: S1 Table — (DOCX) [file pone.0244925.s001.docx]

S1 Table: Knowledge of respondents about transmission of the virus that causes COVID-19 (Q1_1 through Q1_10).

|  | True | False | Don't know | Total |
| --- | --- | --- | --- | --- |
|  | Row N % | Row N % | Row N % | Row N % |
| The virus that causes COVID-19 spreads via respiratory droplets | 92.0% | 5.4% | 2.6% | 100.0% |
| The virus that causes COVID-19 is thought to spread mainly from person to person | 92.9% | 6.2% | .9% | 100.0% |
| An individual who had the disease can spread the illness to others? | 99.2% | .7% | .1% | 100.0% |
| An individual in quarantine can spread the illness to others? | 35.1% | 63.0% | 1.9% | 100.0% |
| The virus that causes corona (COVID-19) can spread through surfaces | 34.0% | 48.9% | 17.0% | 100.0% |
| Infected people cannot transmit the virus if they do not exhibit fever | 90.5% | 7.4% | 2.1% | 100.0% |
| Warm weather would stop the outbreak | 46.9% | 39.9% | 13.2% | 100.0% |
| There is no effective cure for COVID-19 | 28.0% | 66.8% | 5.1% | 100.0% |
| The virus that causes COVID-19 spreads via respiratory droplets | 43.6% | 43.2% | 13.2% | 100.0% |
| The virus that causes COVID-19 is thought to spread mainly from person to person | 86.4% | 11.8% | 1.8% | 100.0% |
