# Supplementary material for: Knowledge, attitudes and practices (KAP) towards COVID-19 among Palestinians during the COVID-19 outbreak: A cross-sectional survey
Source: PLoS One. 2021 Jan 5;16(1):e0244925. doi: 10.1371/journal.pone.0244925 (PMC7785223; doi:10.1371/journal.pone.0244925)
Supplement: S2 Table — (DOCX) [file pone.0244925.s002.docx]

S2 Table: Knowledge of respondents about Covid-19 symptoms (Q1_11_1 through Q1_11_9)

|  | True | False | Don't know | Total |
| --- | --- | --- | --- | --- |
|  | Row N % | Row N % | Row N % | Row N % |
| Fever | 98.2% | 1.4% | .4% | 100.0% |
| Sneezing | 82.6% | 15.9% | 1.5% | 100.0% |
| Dry cough | 95.1% | 3.5% | 1.4% | 100.0% |
| Vomiting | 44.9% | 42.6% | 12.5% | 100.0% |
| Runny nose | 52.7% | 39.6% | 7.7% | 100.0% |
| Blurred vision | 30.8% | 47.4% | 21.8% | 100.0% |
| Mylgia | 73.3% | 18.4% | 8.3% | 100.0% |
| Stuffy nose | 58.2% | 30.8% | 11.0% | 100.0% |
| Frequent urination | 12.1% | 57.4% | 30.5% | 100.0% |
