# Supplementary material for: Knowledge, attitudes and practices (KAP) towards COVID-19 among Palestinians during the COVID-19 outbreak: A cross-sectional survey
Source: PLoS One. 2021 Jan 5;16(1):e0244925. doi: 10.1371/journal.pone.0244925 (PMC7785223; doi:10.1371/journal.pone.0244925)
Supplement: S3 Table — (DOCX) [file pone.0244925.s003.docx]

S3 Table: Knowledge of respondents about high risk groups (Q1_12_1 through Q1_12_9)

|  | True | False | Don't know | Total |
| --- | --- | --- | --- | --- |
|  | Row N % | Row N % | Row N % | Row N % |
| Diabetics | 93.0% | 5.1% | 1.8% | 100.0% |
| People with respiratory illness | 98.8% | .7% | .5% | 100.0% |
| People with heart condition | 93.8% | 4.1% | 2.1% | 100.0% |
| The elderly (70 years or more) | 97.4% | 2.0% | .6% | 100.0% |
| People at age category (50 to 69 years old) | 74.1% | 22.1% | 3.8% | 100.0% |
| People at age category (30 to 49 years old) | 25.2% | 70.0% | 4.7% | 100.0% |
| Young Adults (20-29 years old) | 16.5% | 80.8% | 2.7% | 100.0% |
| Teenagers (10-20 years old) | 19.5% | 77.4% | 3.2% | 100.0% |
| Children under 10 years old are in high risk categories | 41.2% | 55.1% | 3.8% | 100.0% |
