# Supplementary material for: Knowledge, attitudes and practices (KAP) towards COVID-19 among Palestinians during the COVID-19 outbreak: A cross-sectional survey
Source: PLoS One. 2021 Jan 5;16(1):e0244925. doi: 10.1371/journal.pone.0244925 (PMC7785223; doi:10.1371/journal.pone.0244925)
Supplement: S4 Table — (DOCX) [file pone.0244925.s004.docx]

S4 Table: Knowledge of respondents about preventative measures (Q1_13 through Q1_18)

|  | True | False | Don't know | Total |
| --- | --- | --- | --- | --- |
|  | Row N % | Row N % | Row N % | Row N % |
| Individuals can wear masks to prevent the infection by the COVID-19 virus | 70.6% | 28.3% | 1.1% | 100.0% |
| It is not necessary for children and young adults to take preventative measures against the virus | 14.8% | 84.9% | .3% | 100.0% |
| individuals should avoid going to crowded places as a preventative measure against the virus | 97.8% | 2.2% | 0.0% | 100.0% |
| Isolation is an effective way to reduce the spread of the virus. | 97.9% | 1.7% | .3% | 100.0% |
| An individual who has been in close contact with an infected person should be in quarantine for 14 days | 90.8% | 9.0% | .2% | 100.0% |
| COVID-19 is caused by a coronavirus called SARS-CoV-2 | 48.2% | 25.4% | 26.5% | 100.0% |
