# Supplementary material for: Knowledge, attitudes and practices (KAP) towards COVID-19 among Palestinians during the COVID-19 outbreak: A cross-sectional survey
Source: PLoS One. 2021 Jan 5;16(1):e0244925. doi: 10.1371/journal.pone.0244925 (PMC7785223; doi:10.1371/journal.pone.0244925)
Supplement: S5 Table — (DOCX) [file pone.0244925.s005.docx]

S5 Table: Practices of respondents towards preventative measures (Q3_8 through Q3_12).

|  | Yes | No | Total |
| --- | --- | --- | --- |
|  | Row N % | Row N % | Row N % |
| During the last week, have you been in a crowded place? | 15.5% | 84.5% | 100.0% |
| During the last week, have you been to your work place? | 14.3% | 85.7% | 100.0% |
| During the last week, have you visited your neighbors? | 18.8% | 81.2% | 100.0% |
| During the last week, have you visited any of your relatives and/or friends? | 37.6% | 62.4% | 100.0% |
| During the last week, have you worn gloves? masks when leaving home? | 39.9% | 60.1% | 100.0% |
| During the last week, have you kept a distance of at least two between you and others? | 85.2% | 14.8% | 100.0% |
| During the last week, have you cleaned your hands regularly by rubbing an alcohol-based hand sanitizer or washing thm with soap and water? | 96.9% | 3.1% | 100.0% |
